# Extraction of magnetic circular dichroism effects from blended mixture of magnetic linear dichroism signals in the cobalt/Scotch tape system

**DOI:** 10.1038/s41598-019-53880-1

**Published:** 2019-11-20

**Authors:** Chien-Hua Huang, Hua-Shu Hsu, Shih-Jye Sun, Yu-Ying Chang, Paweł Misiuna, Lech Tomasz Baczewski

**Affiliations:** 1 Department of Applied Physics, National Pingtung University, 4-18, Minsheng Road, Pingtung, 90044 Taiwan, ROC; 2 Department of Applied Physics, National University of Kaohsiung, 700, Kaohsiung University Road, Kaohsiung, 81148 Taiwan, ROC; 3 Institute of Physics Polish Academy of Sciences, Al. Lotnikow 32/46, 02-668 Warszawa, Poland

**Keywords:** Condensed-matter physics, Magnetic properties and materials

## Abstract

Circular dichroism (CD) signals revealed in some materials may arise from different origins during measurements. Magnetic field dependent CD (MCD) emanating from the spin-polarized band provides direct insight into the spin–spin interband transitions in magnetic materials. On the contrary, natural CD effects which are artefactual signals resulting from the linear polarization (LP) components during the polarization modulation with a photo-elastic modulator in anisotropic polymer systems were usually observed. There is no simple method to reliably distinguish MCD effect due to spin polarized band structures from natural CD effect, which limits our understanding of the magnetic material/polymer hybrid structures. This paper aims to introduce a general strategy of averaging out the magnetic linear dichroism (MLD) contributions due to the anisotropic structure and disentangling MCD signal(s) from natural MCD signal(s). We demonstrate the effectiveness of separating MCD from natural MCD using rotational MCD measurement and presented the results of a sample with Co thin film on polymer Scotch tape (unplasticized polyvinyl chloride) glued on a quartz substrate. We demonstrate that the proposed method can be used as an effective tool in disentangling MCD and natural MCD effects, and it opens prospects to study the magnetic material /polymer hybrid systems.

## Introduction

Magneto-optics is a useful method to detect electronic structures in magnetic and nonmagnetic materials. Magnetic circular dichroism (MCD) measures the absorption difference of a sample for the left and the right circularly polarized (LCP and RCP) light in the presence of a magnetic field, which is oriented in parallel with the direction of light propagation^1–4^. MCD spectra can be used to study transitions that are difficult to detect by ordinary optical absorption spectroscopy due to their weak excitation coefficients. Because the metals with degenerate energy levels usually induce strong MCD signals, paramagnetic properties and the electronic levels in the systems with metal ion sites are suitable for MCD measurements^5–8^. In addition, MCD spectra can also be a powerful tool to determine both spin and oxidation states for ferric heme proteins systems^6,7^. The direct observation of the d–d transitions by the MCD spectra contributes to clarify the electronic structures of ferric heme proteins systems.

In the field of solid-state physics, MCD can be used to determine the absorption difference between spin channels to resolve the polarized valence states in magnetic materials^9–17^. This technique is particularly sensitive to the polarized electronic states near the band edge. Recently, magneto-plasmonic modes in colloidal gold and silver nanoparticles were also observed using MCD spectroscopy, providing a conceptual proof for possible implementation of magneto-plasmonic refractometric sensing^18–21^. Han *et al*. provided a comprehensive review of the MCD measurements in nanomaterials which provide new opportunity in understanding modulation of excitonic and plasmonic resonances^22–24^. These reports show that MCD measurements, which has been developed for several decades, still plays an important role in various modern research fields.

However, in some natural optical activity, such as chiral molecules, the difference between the LCP and the RCP lights can also be caused by the asymmetry of the molecules^25–28^. For example, due to the handedness of a particular molecule, the absorption spectrum of LCP light is different from that of RCP light. However, polymer materials, as well as anisotropic crystalline with different refraction indexes along different axes, induce the linear birefringence (LB) and linear dichroism (LD) effects. Polarization modulation method with a photo-elastic modulator (PEM) through linear polarization (LP) is commonly used to modulate the LCP and RCP continuously in a commercial CD spectrometer. Therefore, unless the sample is isotropic, the commingling of “natural CD” signals arising from LB and LD need to be considered carefully^29–31^. The complete elimination of the LP component effect by acquiring the difference between the transmitted light intensities measured separately for LCP and RCP incidence could be achieved by the discrete illumination of circularly polarized light^31,32^. In addition, the LD and LB contribution could be cancelled out if the LP components were rotated with time by using a spinning half waveplate^31,33^. Besides, if the sample is uniform over the area of observation, to rotate the anisotropic samples in the observing plane (normal to the direction of light incidence) sometimes can be expected to suppress the LP component influence^31^.

In general, the signal intensities of CB and CD involving an optical response to circular polarization are usually two or three orders of magnitude smaller when compared with those of LB and LD which originate in the anisotropy of materials against linear polarization^31,34,35^. However, nowadays, hybrid polymer and magnetic materials have attracted increasing research attention in the area of organic spintronics and the interaction between magnetism and molecular chirality^26–28,36–38^. It is necessary to clarify the issue mentioned previously when taking the investigation of magnetic field effect on natural CD measurement. Sometimes the intensity of MCD signal from magnetic material is similar to the intensity of natural MCD from LP components. Therefore, the interpretations of MCD signals in hybrid polymer/magnetic materials need to be carefully checked. One fundamental question still remaining is that can the MCD from spin polarized bands and natural MCD effects be separated using a simple method during CD measurements under applied magnetic fields?

In this work, we have constructed a rotational holder fixture applied for rotational MCD measurements. We show the results of MCD measurements on the sample composed of 50 nm thick polycrystalline Co thin film deposited on polymer Scotch tape glued on quartz substrate. It is confirmed that the proposed rotational MCD method is efficient in the extraction of MCD effects from blended mixture of magnetic LD (MLD) signals in the cobalt/Scotch tape system.

## Results and Discussion

**Figure 1:**
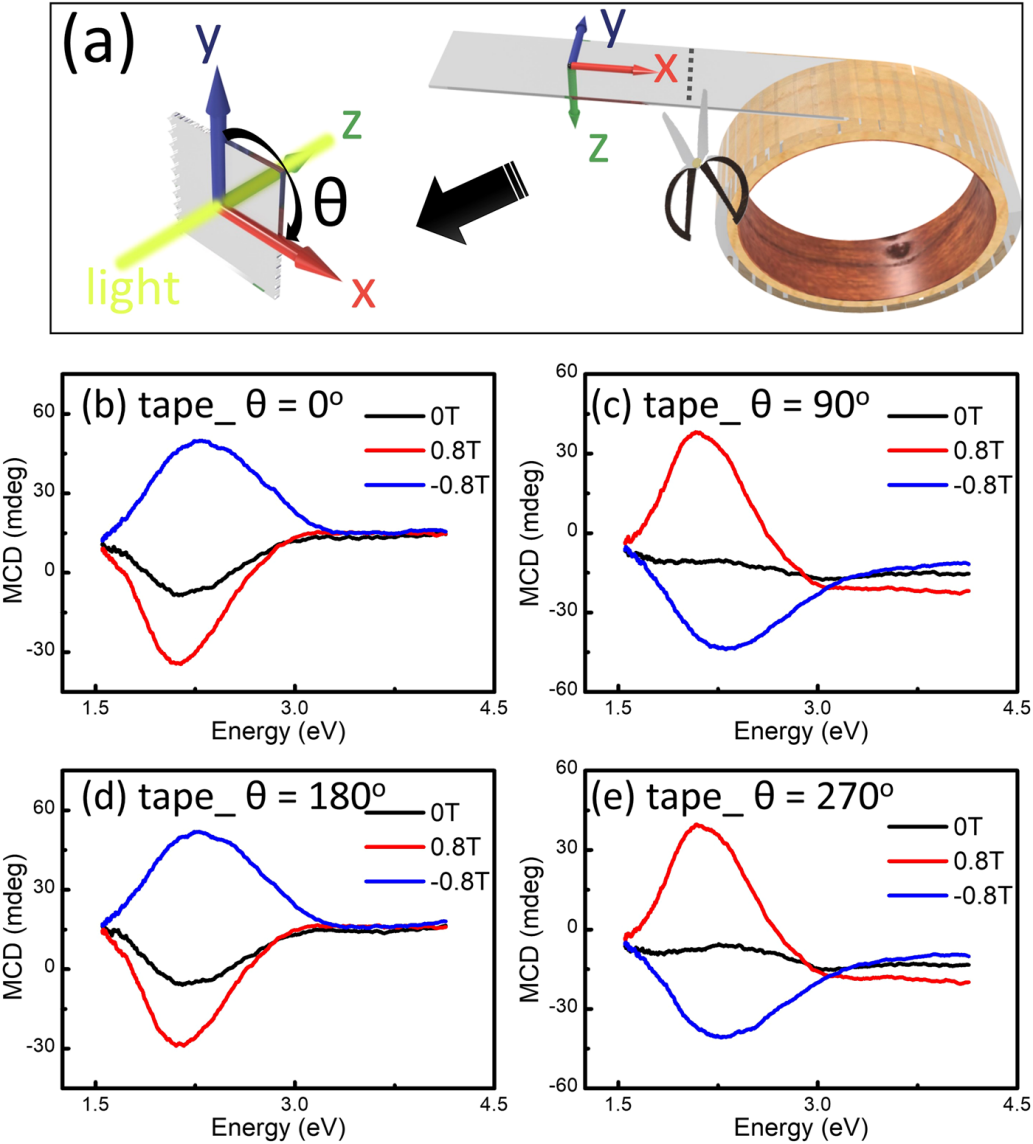
(**a**) Schematic illustration of the experimental setup of MCD measurement at different θ. Light propagates along the +z axis and is parallel to the applied magnetic field. The rotation angle (θ) is the azimuth angle with respect to z axis of the light propagation and is defined as the angle between the y axis (0°). MCD spectra of the Scotch tape obtained at (**a**) 0°, (**b**) 90°, (**c**) 180°, and (**d**) 270°.

**Figure 2:**
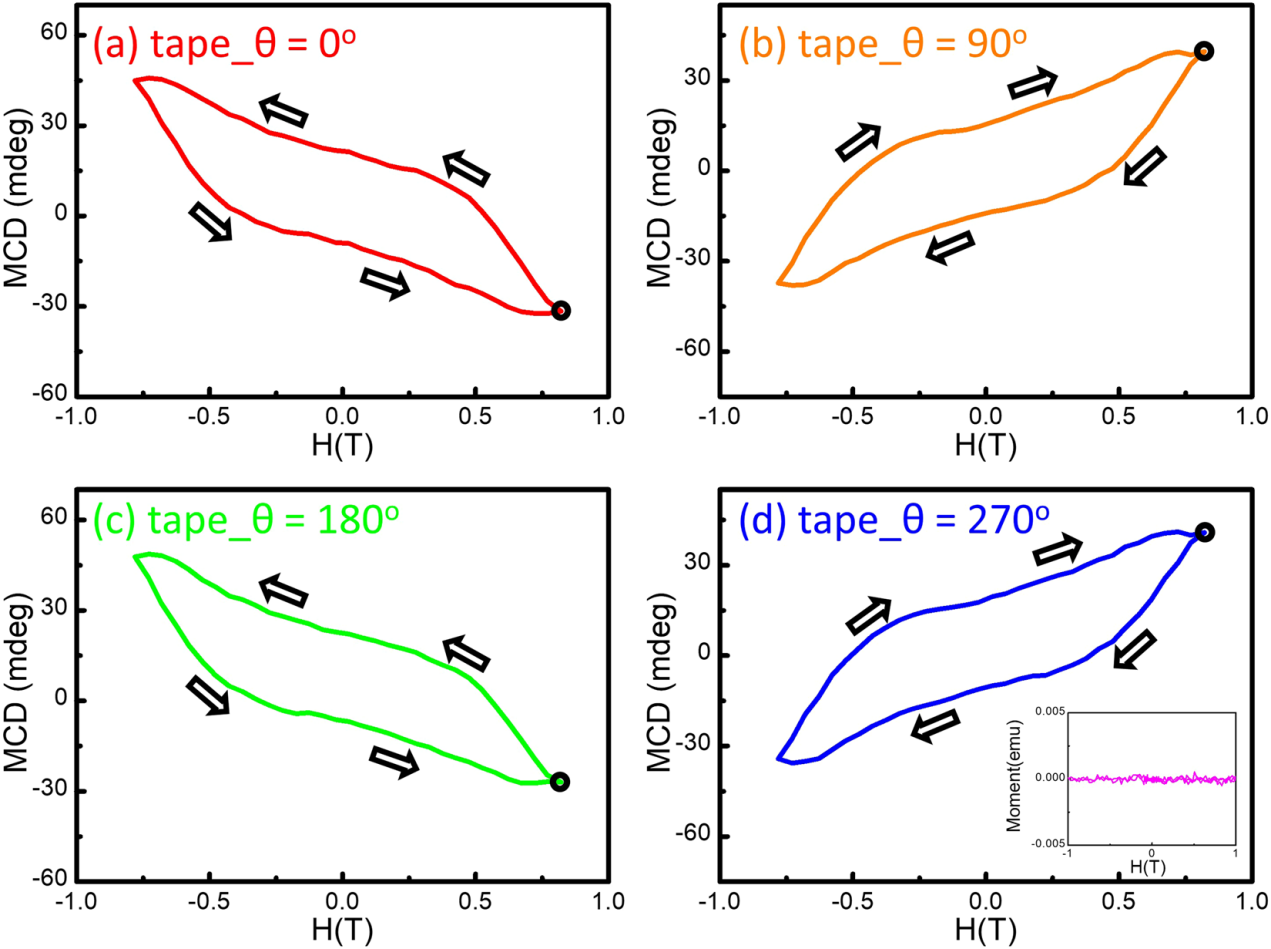
The MCD-H at the 2.1 eV from the Scotch tape obtained at (**a**) 0°, (**b**) 90°, (**c**) 180°, and (**d**) 270°. The inset in (**d**) shows the M-H result of the Scotch tape measured by VSM. No detectable FM was observed.

The Scotch tape has been known to be anisotropic and its refraction indexes are different along x and y axes, as shown in the Fig. 1(a). The rotation angle (θ) is the azimuth angle with respect to z axis of the light propagation. As a result, an artefactual natural CD signal resulting from the LP components is expected. Figure 1(b–e) show the “MCD” spectra (actually natural MCD) of the Scotch tape obtained at 0°, 90°, 180°, and 270°. All spectra were subtracted from the background data including the substrate to give absolute spectra of the tape only. Broad and large natural CD signals were observed. The CD signals reveal the sign change when the sample is rotated by 90° and are similar when the sample is rotated by 180° which indicates the uniaxial anisotropy feature of the tape. The natural CD is also affected by the magnetic field (H), but it remains asymmetric when the magnetic direction changes. We have also performed MCD-H measurements as a function of magnetic field as well as for the Scotch tape at 4 different angles. The MCD-H curves of such signals are not typical MCD signals from magnetic materials as shown in the Fig. 2(a–d). We have also noted that some polymers samples could show room temperature ferromagnetism (FM) after stretching or cutting and the appearance of FM is sensitive to environment, such as humidity. The inset of Fig. 2(d) shows the M-H result of tape measured by vibrating sample magnetometer (VSM). No detectable FM was observed.

In general, the polymers are constructed by polymer chains which are entangled with each other, leading to amorphous structure and average symmetry. However, the polymer chain shows a uniaxial anisotropy hence it has a preferred orientation. This anisotropy will appear when a tension force is applied along the preferred orientation. It can be imagined that the Scotch tape should have a tension force along the tape longitudinal direction during its fabrication and induce different refraction indexes along x and y axes. Due to the existence of the LD effect in the tape, the natural MCD effect will be induced expectedly.

**Figure 3:**
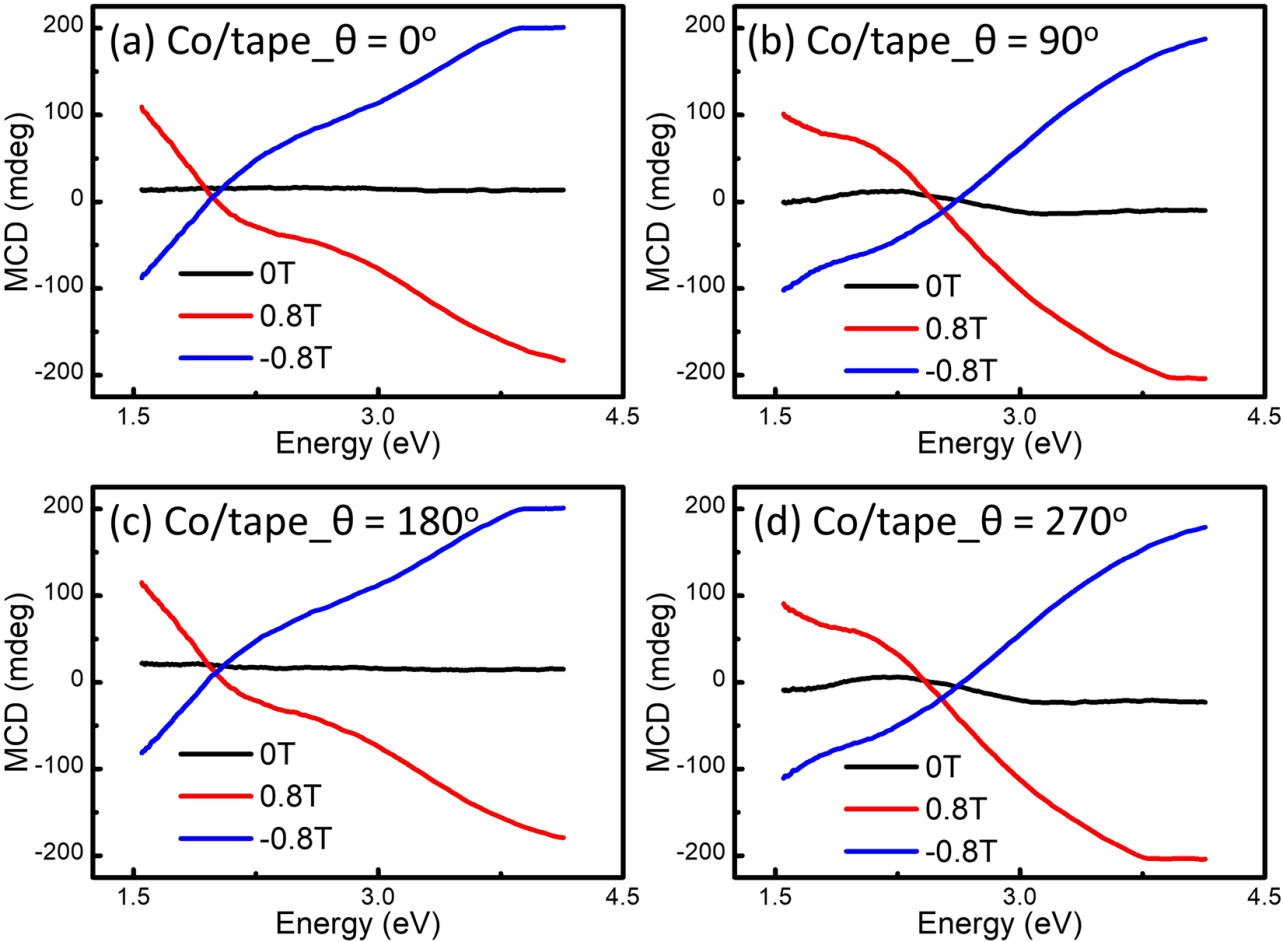
The MCD spectra from Co 50 nm/Scotch tape obtained at (**a**) 0°, (**b**) 90°, (**c**) 180°, and (**d**) 270°.

**Figure 4:**
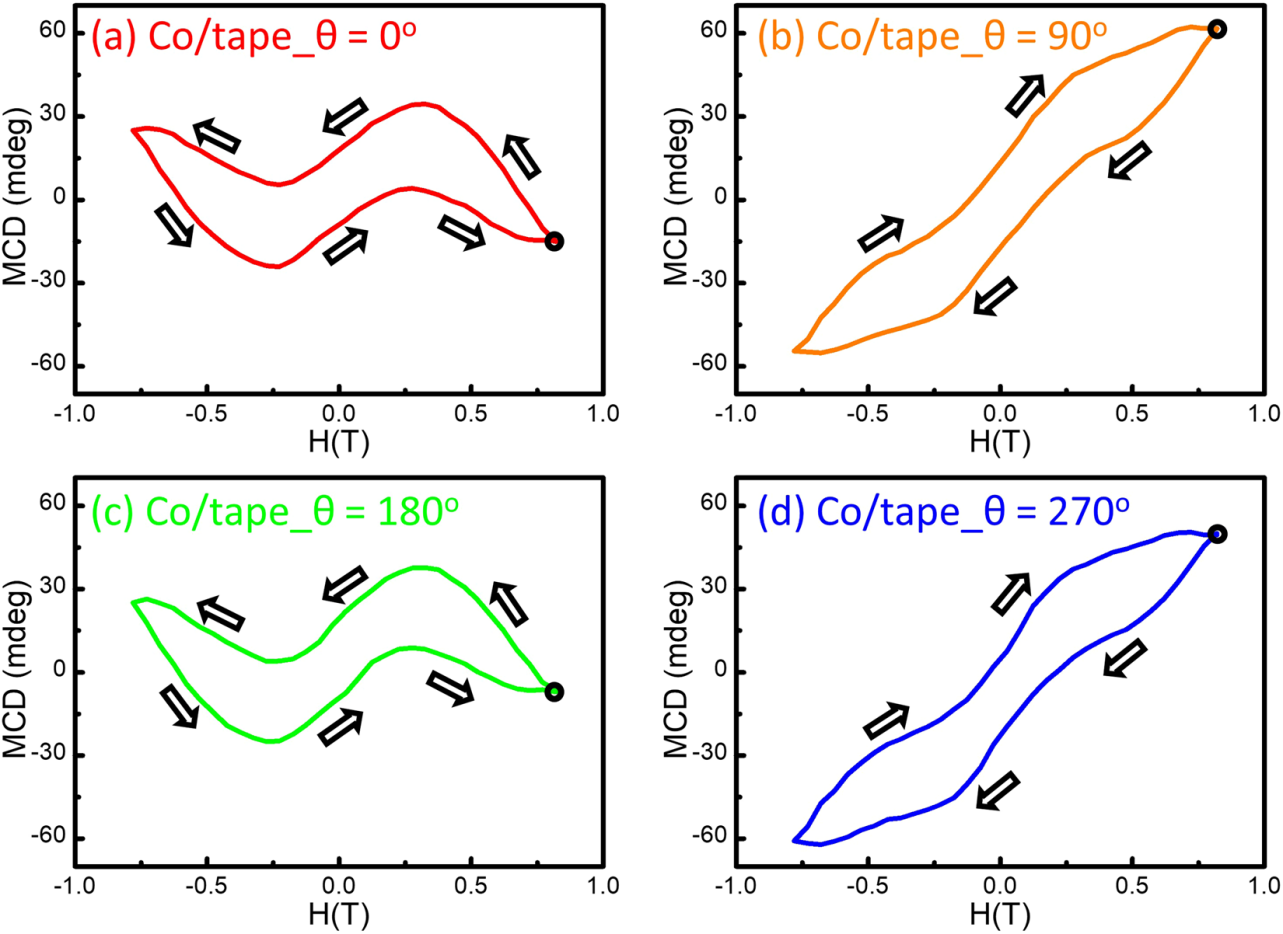
The MCD-H at the 2.1 eV from Co 50 nm/Scotch tape obtained at (**a**) 0°, (**b**) 90°, (**c**) 180°, and (**d**) 270°.

The Co/tape film sample was also measured using the procedure mentioned above. The MCD spectra from Co and natural CD from polymers, are superimposed on each other, as shown in Fig. 3(a–d). In particular, the MCD-H effect at 2.1 eV shows unusual loops which are hard to understand and interpret, see Fig. 4(a–d). Therefore, the origin of these “MCD” strange loops observed in such hybrid systems should be carefully studied.

**Figure 5:**
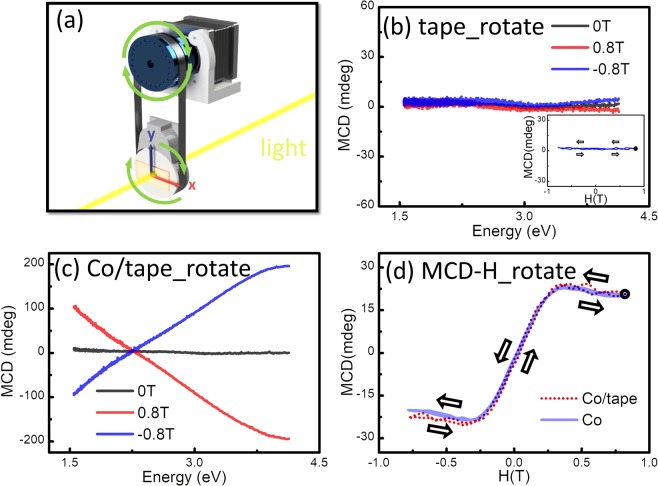
(**a**) A schematic pattern of rotational holder fixture. (**b**) The MCD spectra of the Scotch tape with the rotational fixture. The inset in (**b**) is the MCD- H curve of Scotch tape for rotational measurement. (**c**) The MCD spectra from Co 50 nm/polymers (Scotch tape). (**d**) The MCD-H loop at 2.1 eV of the 50 nm Co film on quartz substrate and Co 50 nm/ Scotch tape measured by rotational holder fixture.

As mentioned above, the polymer with a uniaxial anisotropy exhibits different refraction indexes along x and y axes, therefore, resulting in a θ dependent MCD signal. If we sum all the measured “MCD” signals from θ = 0° to 360°, the interference due to the natural CD signal will be thus eliminated. To overcome this limitation and eliminate natural MCD effect, a rotational holder fixture has been designed to separate these two kinds of MCD signal. A schematic view of our rotational fixture is shown in Fig. 5(a). A motor was used to rotate the sample holder with a rotating speed of 0.5 rps. The integration time per data point is 4 s. This means every data point was collected and averaged over a rotation of 2 circles.

**Figure 6:**
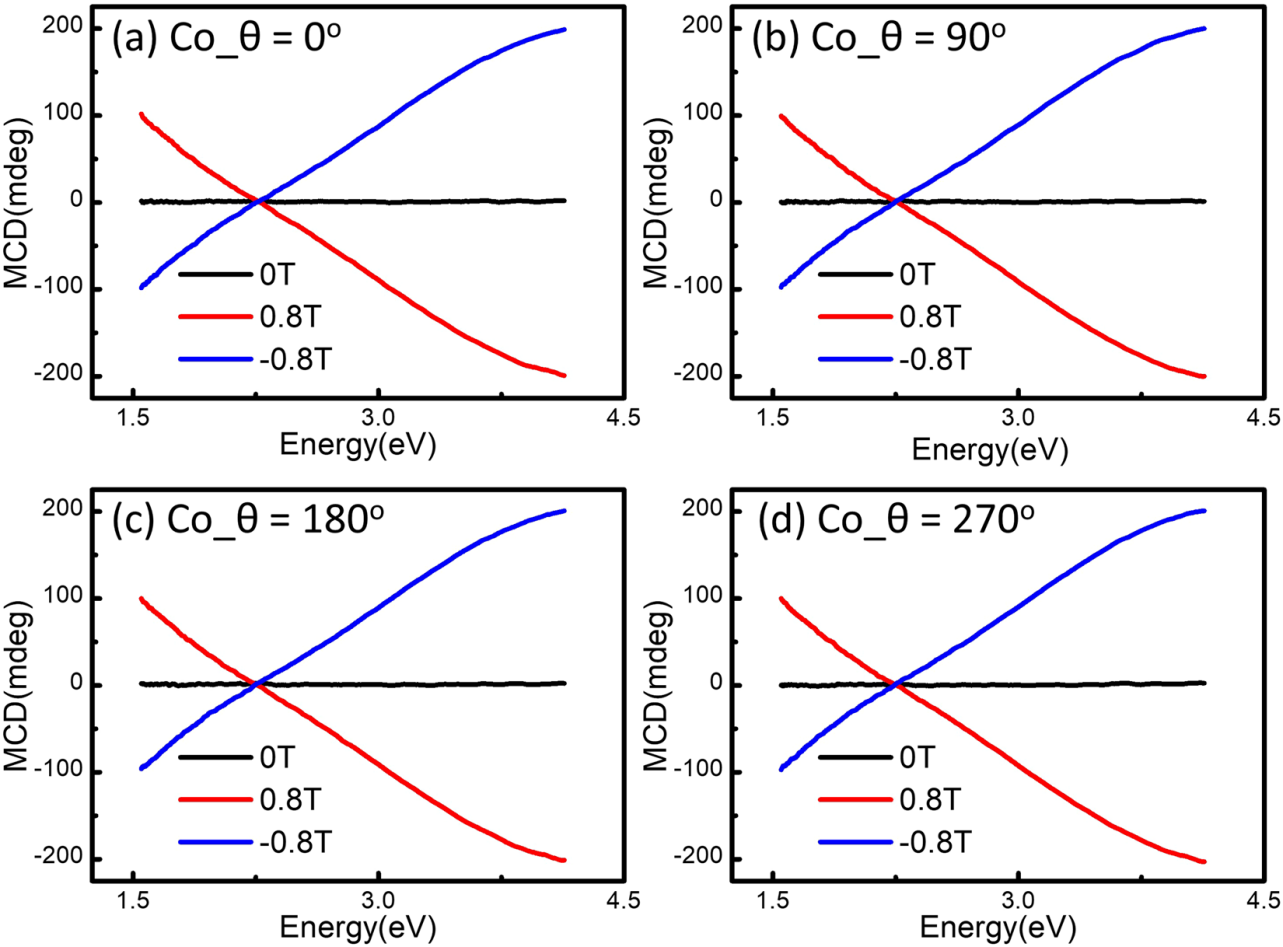
The MCD spectra from only-Co obtained at (**a**) 0°, (**b**) 90°, (**c**) 180°, and (**d**) 270°.

**Figure 7:**
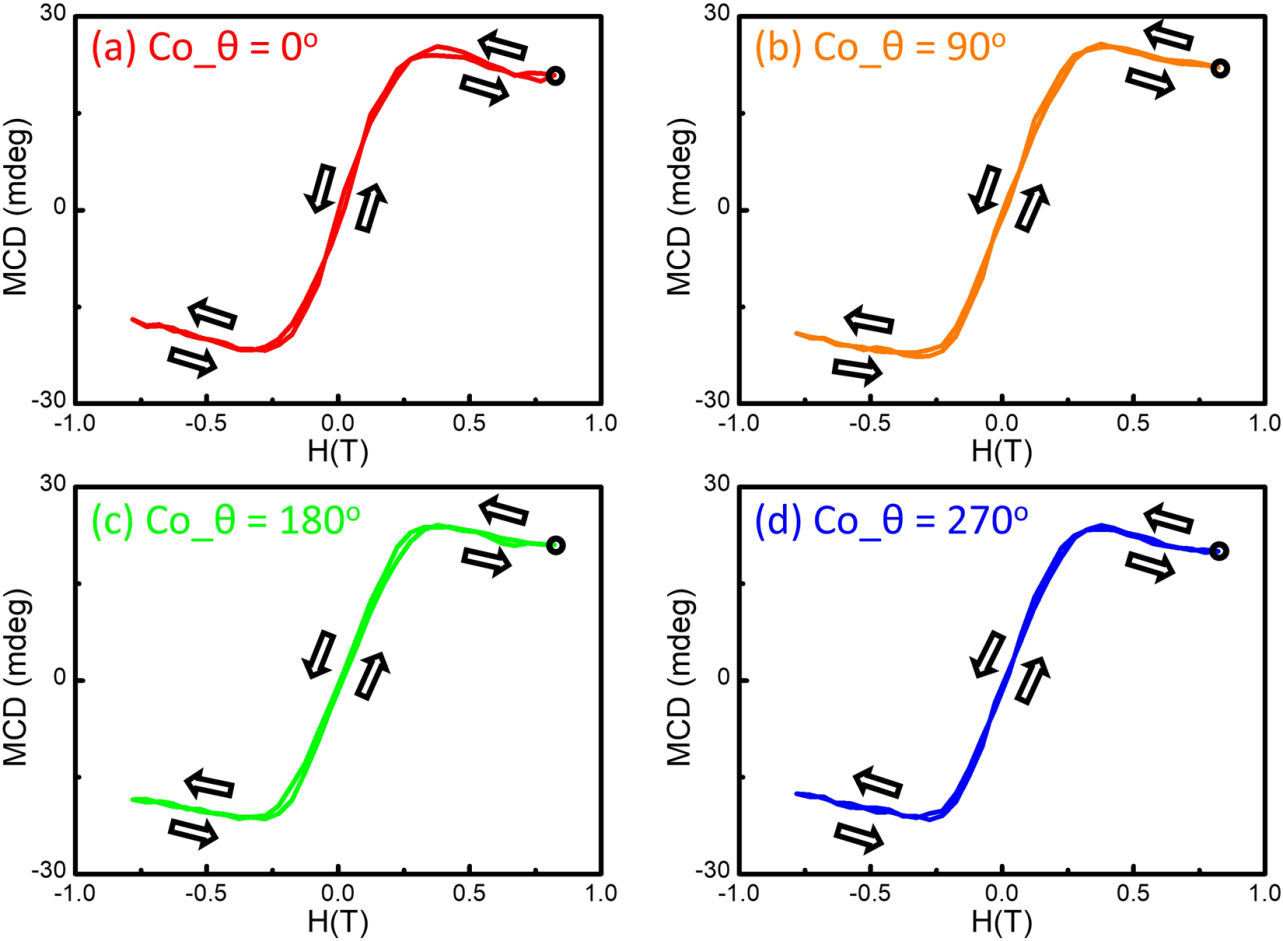
The MCD-H at the 2.1 eV from the only-Co obtained at (**a**) 0°, (**b**) 90°, (**c**) 180°, and (**d**) 270°.

Figure 5(b) shows as a reference of the MCD spectra and the MCD-H curve of the Scotch tape with the rotational holder fixture. It is clear that the natural MCD signal due to the anisotropic structure has been eliminated as expected. The averaged signals behave like flat backgrounds and are not affected by the applied magnetic field. Therefore, Fig. 5(c,d) display the MCD spectra respectively, and MCD-H hysteresis loop at 2.1 eV of the Co/tape sample measured using the rotational holder fixture. Due to the rotational averaging, the natural MCD signal has been successfully removed. The MCD result is in a good agreement with the one for pure Co thin films on quartz substrate where the MCD effect of Co film shows no angular dependence. Because the MCD signals consist of the sum of magnetic and intrinsic Zeeman contributions, the magnetic contribution becomes field-independent when all spins have been aligned by the magnetic field, but the intrinsic Zeeman contributions continues to increase linearly with H. Therefore, the MCD-H curves for Co film and Co/tape are titled at high magnetic field^10,22^. The results are consistent with the MCD spectra and MCD-H hysteresis loop at 2.1 eV of only-Co at 4 angles, as displayed in the Figs 6(a–d) and 7(a–d), respectively. The strategy provided here permits the MCD investigation for magnetic material/polymer hybrid heterostructures and enables to probes the interfacial interaction and other phenomena emerging at interfaces in the future.

**Figure 8:**
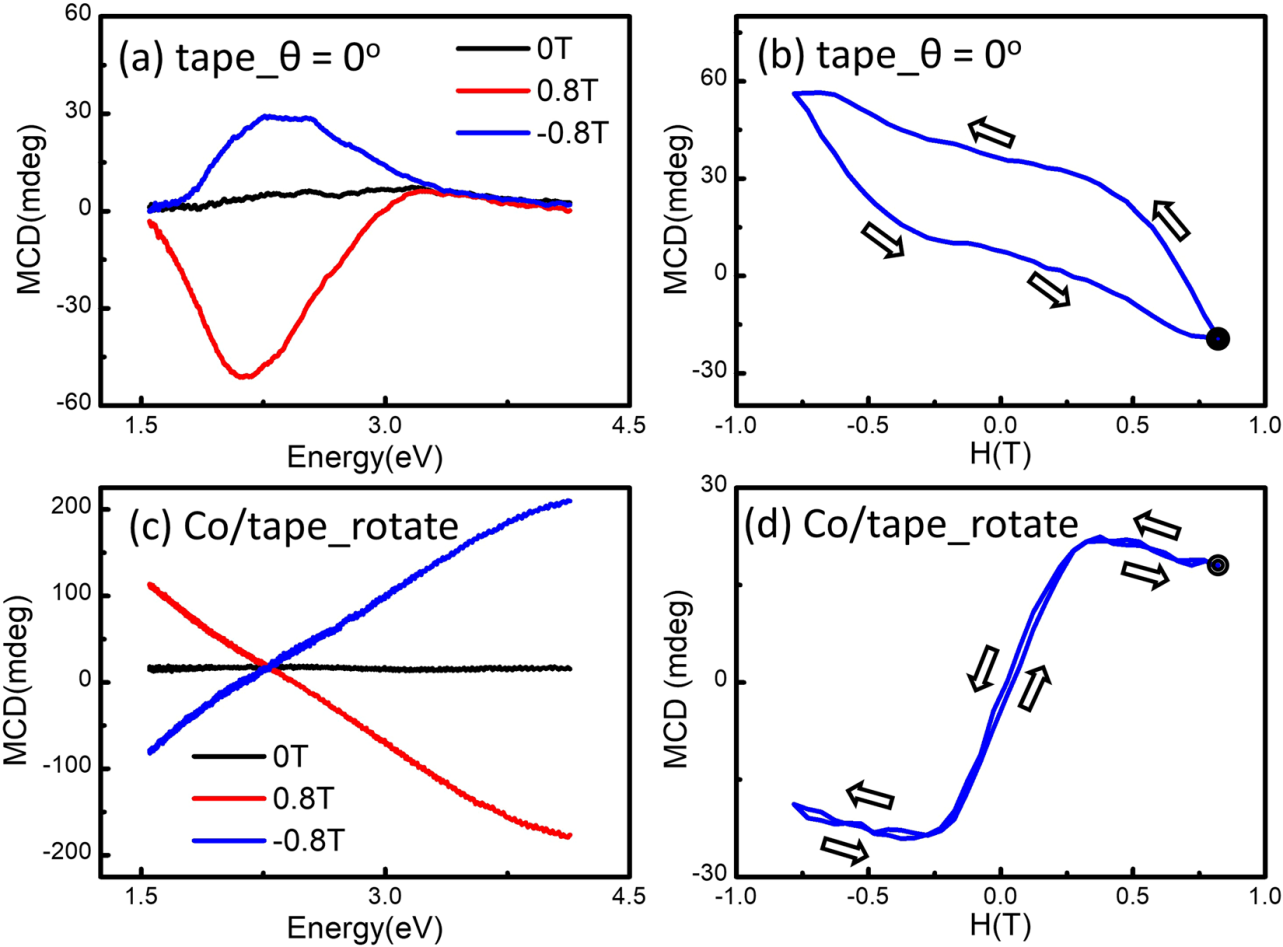
(**a**) The MCD spectra and (**b**) the MCD-H at 2.1 eV of the Scotch tape obtain from different position at 0°. (**c**) The MCD spectra and (**b**) the MCD-H loop at 2.1 eV of the Co 50 nm/Scotch tape measured by rotational holder fixture from different position. The nonuniformity of the tape indeed results in inhomogeneous natural MCD effect in different position but the rotational MCD measurement will remedy the nonuniformity of tape could extract the intrinsic MCD signals of Co to avoid the positioning issue.

In addition, although one can remove the MCD effect of tape from the MCD spectra of Co/tape by subtraction procedures, positioning the light spot carefully during the measurements of MCD effects of tape and magnetic material/tape is crucial because of the nonuniformity of tape which will induce inhomogeneous natural MCD effect at different positions. For example, the curve in Fig. 8(a) is different from the curve in Fig. 1(a) and the curve in Fig. 8(b) is different from that in Fig. 2(a), due to the nonuniformity of tape. The rotational MCD measurement is providing a remedy to the nonuniformity of tape by averaging out the MLD. Therefore, Fig. 8(c,d) are almost identical to Fig. 5(c,d), respectively. These results further prove that the rotational MCD measurement is a simple but effective method to extract the intrinsic MCD signals of magnetic material from anisotropic natural MCD signals of tape to avoid the positioning issue.

On the other hand, using a primitive “Scotch” tape has been considered as the simplest mechanical cleavage technique which has resulted in the Nobel-awarded discovery of graphenes and is currently under worldwide use for assembling graphenes and other two-dimensional (2D) structures^39–45^. To extract the MCD signal from Scotch tape using the proposed here a rotational holder fixture for rotational MCD measurements will open a possibility to investigate the magnetic 2D material directly on tape after mechanical cleavage without additional transfer process.

Besides, molecular and polymeric ferromagnets have been intensively studied in the past three decades^46–51^, room temperature FM due to carbon dangling bonds without introducing transition metal elements has also been demonstrated in polymers and carbon-based molecular materials through cutting and stretching^51^. It is expected in our own method that investigation of the intrinsic MCD effect from spin polarized band of polymer can be achieved and free from the natural MCD signals.

## Conclusion

In summary, we have constructed and successfully applied a rotational holder fixture for MCD measurements of magnetic material/polymer structures. From the MCD measurements of Co/polymer thin films, it is confirmed that the rotational MCD measurement is an effective way to separate the MCD signal caused by spin-polarized band from the natural CD caused by polymer anisotropy. The pure reference Co thin film MCD result and the Co/polymer thin film rotational MCD one are in good agreement to support the credence of the presented method. Clearly, the effectiveness of this method depends on the structural and spin polarization properties of the polymers and magnetic materials. And it may vary from system to system. We hope that this work will motivate further exploration of the rotational MCD measurements and lead to the improvement of the effectiveness of this approach.

## Methods

A schematic diagram of the MCD measurement is shown in Fig. S1. MCD results were measured at room temperature using a Jasco J-815 spectrometer equipped with a 450 W Xenon lamp. The white light from the lamp was monochromatized and linearly polarized in a monochromator. This light then passed through a PEM, which acted as a quarter wave plate to convert the linearly polarized light into circularly polarized light. It was also switched with a certain frequency (50 kHz) so that it produced both RCP and LCP for MCD measurement. The MCD signal is collected using a photomultiplier. The integration time per data point is 4 seconds. Light propagates along the +z axis and is parallel to the applied magnetic field direction. The light spot is about 3 mm in diameter during measurements.

Next, a reference sample of 50 nm thick Co thin film was deposited at room temperature on quartz substrates using magnetron sputtering with a base pressure at approximately 1 × 10^−6^ Torr. A Co target (99.99%) was used for the sputtering. The working pressure of the sputter gas (Ar, 99.9995% purity) was 1.3–2.0 mTorr and the applied sputtering power was 100 W. A 3 M™ Scotch® Transparent Film Tape 600 (unplasticized polyvinyl chloride, UPVC) was glued on the back of the quartz substrate as a polymer substrate. Strips of Scotch tape with a typical area of 1 × 1 cm^2^ were used in our experiments. To avoid magnetic contamination, the tape was cut with ceramic scissors. All spectra were subtracted from the background data including the substrate to give absolute spectra of the samples.

## Supplementary information

Supplementary Data
