# Supplementary material for: Extraction of magnetic circular dichroism effects from blended mixture of magnetic linear dichroism signals in the cobalt/Scotch tape system
Source: Sci Rep. 2019 Nov 20;9:17192. doi: 10.1038/s41598-019-53880-1 (PMC6868135; doi:10.1038/s41598-019-53880-1)
Supplement: Supplementary file 1 — Supplementary Data [file 41598_2019_53880_MOESM1_ESM.docx]

Supplementary Information

Extraction of magnetic circular dichroism effects from blended mixture of magnetic linear dichroism signals in the cobalt/Scotch tape system

Chien-Hua Huang^1^, Hua-Shu Hsu ^*1^, Shih-Jye Sun^2^, Yu-Ying Chang^1^, Paweł Misiuna^3^ and Lech Tomasz Baczewski^3^

^1^Department of Applied Physics, National Pingtung University, 4-18, Minsheng Road, Pingtung, 90044, Taiwan, R. O. C.

^2^Department of Applied Physics, National University of Kaohsiung, 700, Kaohsiung University Road, Kaohsiung, 81148, Taiwan, R. O. C.

^3^Institute of Physics Polish Academy of Sciences, Al. Lotnikow 32/46, 02-668 Warszawa, Poland

*email: [hshsu@mail.nptu.edu.tw](mailto:hshsu@mail.nptu.edu.tw)

This file includes:

Supplementary Figures S1


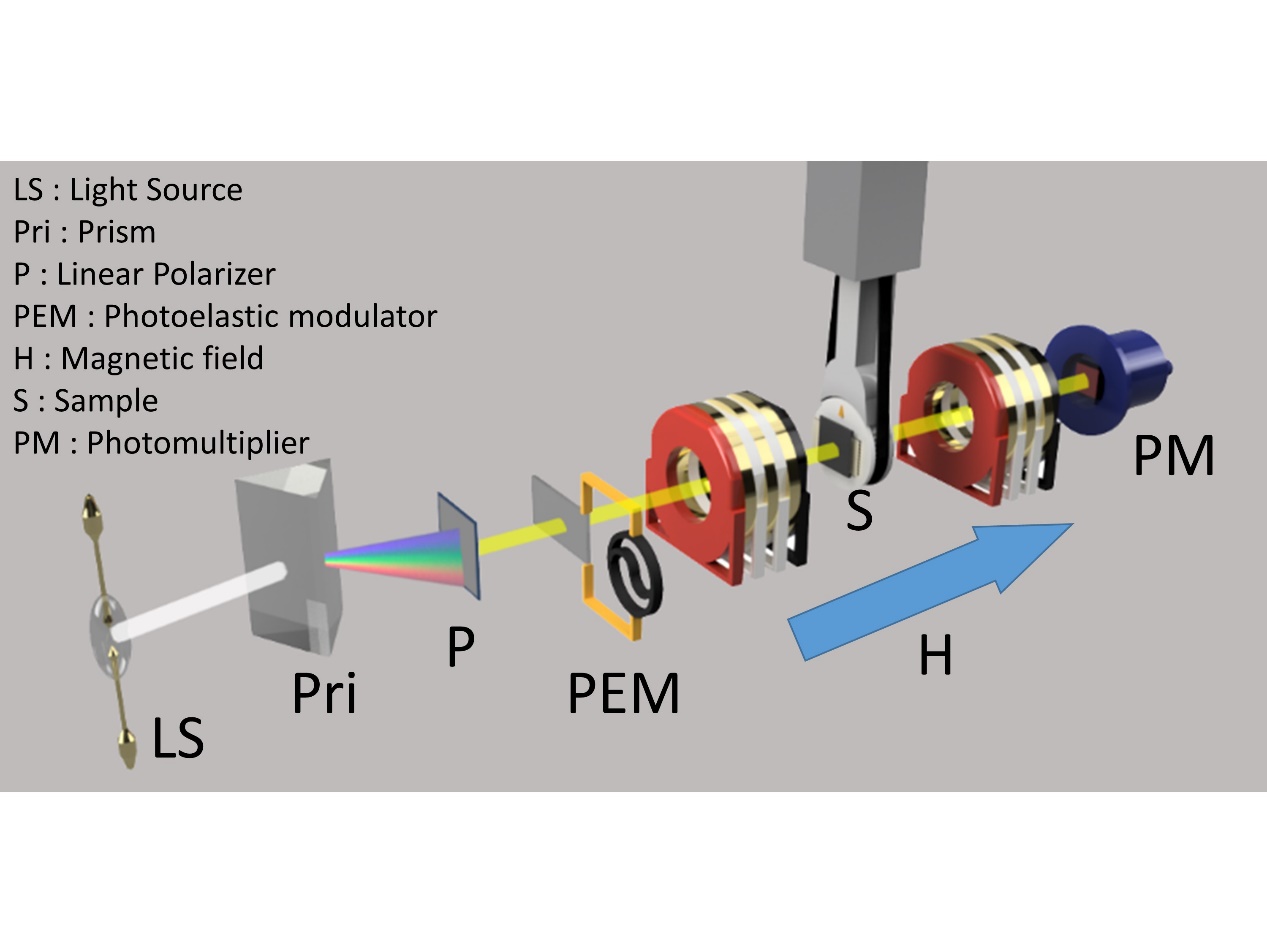
 Figure S1 A schematic diagram of the MCD measurement system.
